# Labile iron starvation in embryonic Kupffer cells aggravates MASH via mitochondrial failure and macrophage dysfunction

**DOI:** 10.1038/s41419-026-08985-7

**Published:** 2026-06-18

**Authors:** Ke Wang, Garam An, Junho Park, Karina Cunha e Rocha, Zixuan Zeng, Houji Qin, Andrea Farrell, Alex Schlotthauer, Whasun Lim, Wei Ying

**Affiliations:** 1 Division of Endocrinology and Metabolism, Department of Medicine, University of California, San Diego, CA USA; 2 School of Biomedical Sciences, Hunan University, Changsha, China; 3 Department of Biological Sciences, College of Science, Sungkyunkwan University, Suwon-si, South Korea; 4 School of Biological Sciences, University of California, San Diego, CA USA

**Keywords:** Immune cell death, Metabolic disorders

## Abstract

Metabolically-dysfunction-associated steatohepatitis (MASH) is characterised by embryonic Kupffer cell (emKC) loss and proinflammatory macrophage infiltration. While iron dysregulation is implicated in MASH, the role of labile iron (Fe2 + ) in mediating emKC survival and function remains unknown. In human and mouse MASLD/MASH livers, emKCs exhibit repressed iron metabolism, reduced labile iron pools, and mitochondrial dysfunction. KC-specific ferroportin knockout mice recapitulate these defects, accelerating Western diet-induced steatosis and fibrosis. Conversely, ferritin depletion restores emKC labile iron levels, mitigates mitochondrial damage, and attenuates disease severity. Fe2+ deficiency blunts Trem2 expression, whereas restoring Fe2+ homeostasis elevates emKC Trem2 abundance in MASH liver. Weight loss reverses hepatic iron dysfunction and restores emKC homeostasis. Our findings establish labile iron deficiency as a key driver of emKC loss and functional switch in MASH, linking iron metabolism to mitochondrial fitness and inflammation. Targeting emKC iron homeostasis could offer novel therapeutic strategies for MASLD/MASH.

## Introduction

Obesity and metabolic dysfunction-associated liver disease (MASLD) are pathophysiologically linked. Driven by the global obesity epidemic, MASLD now affects approximately 20% of the global population, with 30% of these patients progressing to metabolic dysfunction-associated steatohepatitis (MASH), a condition marked by hepatic steatosis, inflammation, and fibrosis [1–3]. As a leading cause of cirrhosis, hepatocellular carcinoma, and liver transplantation, MASH represents a critical public health challenge [4, 5]. Despite advances in understanding disease mechanisms [6–11]. The precise pathogenesis of MASH remains unresolved.

During MASH progression, embryonic Kupffer cells (emKCs), expressing the specific markers CLEC4F and TIM4, undergo progressive death, with ~60% lost by 6 months of Western diet (WD) feeding [12]. A remarkable loss of emKCs is also found in MASLD/MASH human liver [13]. Concurrently, monocyte-derived recruited hepatic macrophages (RHMs) infiltrate the liver, occupying the sinusoidal niches vacated by emKC loss [7, 12, 14, 15]. Single-cell RNA sequencing reveals that these RHMs adopt transcriptional profiles resembling resident KCs [12]. More importantly, shifts in hepatic macrophage populations correlate strongly with MASH pathogenesis [16–20]. emKCs exhibit functional alterations during disease progression [12]. and recent work has expanded our understanding of their role in MASH [21, 22]. For instance, Trem2-expressing hepatic macrophages play a crucial regulatory role in maintaining liver homeostasis, but these macrophages lose Trem2 during MASLD/MASH development, contributing to disease progression [23–27]. However, the mechanisms driving their functional reprogramming remain poorly understood.

The liver is central to systemic iron regulation [28] and emerging evidence links iron homeostasis to metabolic disorders [29–33]. Notably, hepatic iron dysfunction is observed in MASH patients [33–36]. and aberrant iron distribution across hepatic cell types has been implicated in steatosis and fibrosis [37]. Although emKCs are key regulators of hepatic iron, the role of iron metabolism, particularly the labile iron (Fe2 + ) pool, in managing hepatic macrophage function and survival during MASH is unknown [38].

Here, we show that emKCs in MASLD/MASH exhibit repressed iron metabolism and labile iron deficiency, accompanied by mitochondrial defects. Labile iron starvation caused by ferroportin (FPN) depletion repressed mitochondrial functions in emKCs, concomitant with the loss of emKC in lean mice. When challenged with a Western diet, KC-specific *Fpn* knockout (KO) mice developed liver steatosis and fibrosis more quickly than WT littermates. Conversely, depletion of ferritin attenuated MASLD/MASH-related emKC abnormalities and blunted the development of liver steatosis and fibrosis. High levels of labile iron elevated Trem2 expression through a ROS-dependent mechanism. In addition, weight loss efficiently restored the iron metabolism of hepatic macrophages.

## Results

### emKCs are iron-deficient in MASLD/MASH livers

**Fig. 1:**
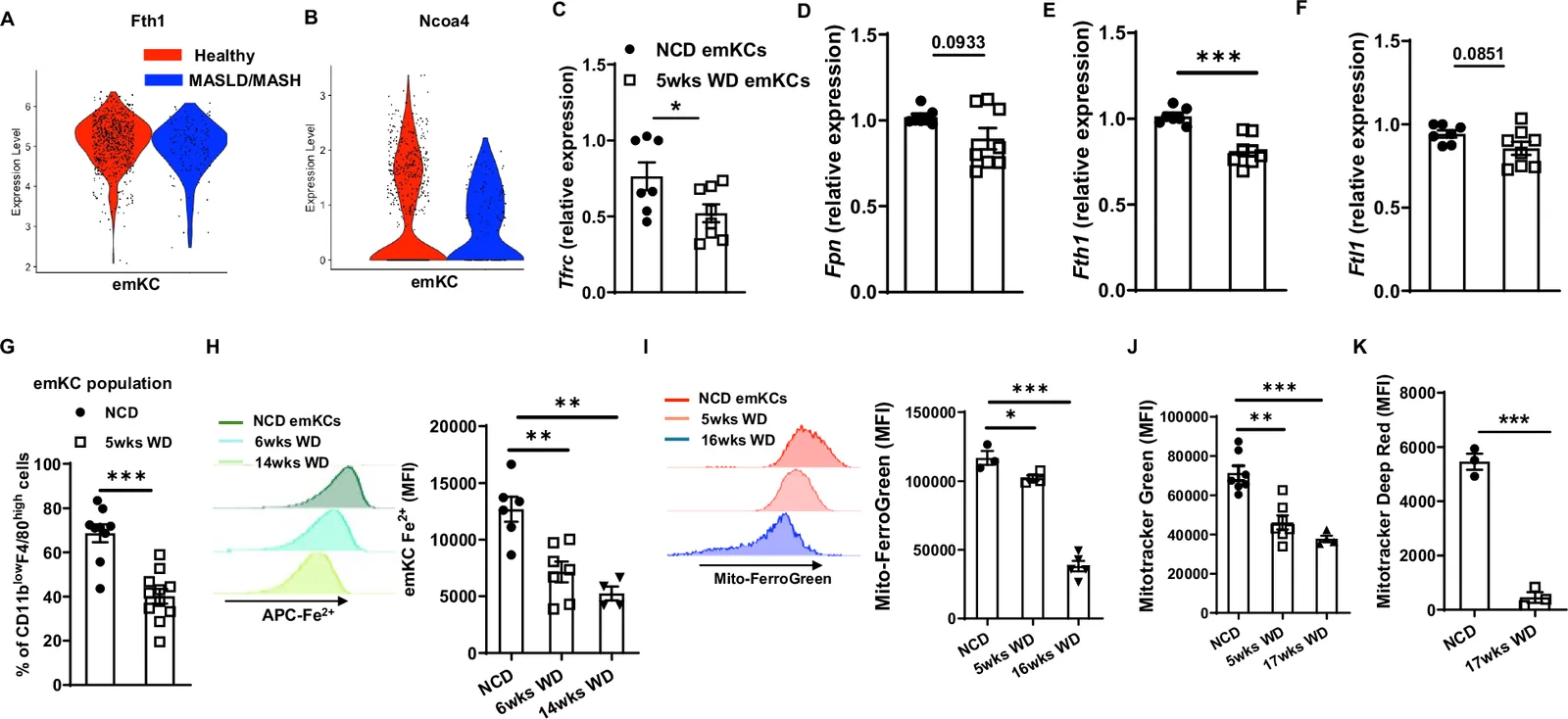
Embryonic Kupffer cell (emKC) iron metabolism in MASLD/MASH liver. The abundance of ferritin (*Fth1*; **A**) and Ncoa4 (**B**) in the emKCs of human livers. The abundance of key genes associated with emKC iron metabolism (**C–F**) and population (**G**) after 5-weeks WD feeding. **H** The abundance of labile iron (Fe2 + ) in emKCs after WD feeding. After WD feeding, mitochondrial Fe2+ levels (**I**), mitochondrial mass (**J**), and mitochondrial membrane activation (**K**) of emKCs were measured by flow cytometric (FACS) analysis with Mito-FerroGreen, Mitotracker Green, or Mitotracker Deep Red probes, respectively. Data are presented as mean ± SEM. *P < 0.05, **P < 0.01, ***P < 0.001, Student’s *t* test (**C–G, K**) or One-way ANOVA (**H–J**).

emKCs are one of the key cell types storing iron and maintaining systemic iron homeostasis [39]. Our recent findings reveal abnormalities in iron distribution among hepatic cells during MASH development [37]. By analyzing the published human liver single-cell RNAseq dataset [13]. We found that MASLD or MASH human livers are characterized by a remarkable reduction in the number of emKCs (highly expressed TIMD4, MARCO, and CD68) [13, 40]. which represses the abundance of key genes associated with iron homeostasis (Fig. S1A). For example, MASLD/MASH human liver emKCs expressed much lower levels of ferritin (*Fth1*) than healthy emKCs, suggesting iron deficiency in MASLD/MASH emKCs (Fig. 1A). In addition, the expression of NCOA4, the key mediator producing labile iron from ferritinophagy [41–43]. was also decreased in MASLD/MASH human emKCs (Fig. 1B).

Consistently, in the Western diet (containing 40% fat calories and 0.2% cholesterol), feeding induced MASLD/MASH mouse model [21, 37]. emKCs (TIM4^+^CLEC4F^+^CD11b^low^F4/80^high^) quickly exhibited impaired iron metabolism, as evidenced by less abundance of transferrin receptor (*Tfrc1*; iron uptake) and ferroportin (*Fpn*; iron export) than NCD emKCs, concomitant with significant loss of emKCs (Fig. 1C–G and S1B). After 16 weeks of WD feeding, emKC iron metabolism abnormalities became worse, and the emKC number was also further reduced (Fig. S1C–G).

Given the reduction in total iron content in obese emKCs, we further assessed the abundance of labile iron, which is essential for various biological activities [44–47]. Using a labile iron fluorescent probe that only reacts with Fe2+ and irreversibly turns it into a far-red fluorescent substance, we found that the levels of labile iron were quickly decreased in emKCs after a short period of WD feeding (Fig. 1H) [48]. Prolonged WD feeding caused a greater reduction in the levels of labile iron pool of emKCs (Fig. 1H). In high-fat diet (containing 60% fat calories)-induced obese mice, emKCs were also labile iron deficient (Fig. S1H). A previous study suggests emKCs ferroptosis in obese livers [49]. In contrast, by analyzing the published RNAseq dataset, the expression of key genes associated with ferroptosis in KCs was not significantly changed in WD-fed mice (Fig. S1I) [12].

### MASH emKCs are characterized by impaired mitochondrial functions

As labile iron is essential for mitochondrial functions, we next examined whether MASLD/MASH-induced labile iron deficiency affects mitochondrial phenotypes in emKCs [44–47]. We used flow cytometric analysis with a fluorescent probe (Mito-FerroGreen) to specifically detect mitochondrial Fe^2+^ which is bound with hemes and iron-sulfur clusters [50]. Concomitant with decreased intracellular labile iron content in response to WD feeding, mitochondrial ferrous iron levels were also quickly reduced after a short-term (5-weeks) WD feeding and markedly decreased after a chronic long-term (16 weeks) WD feeding (Fig. 1I). Consistently, the emKCs of WD-fed mice displayed impaired mitochondrial functions, as shown by dramatically decreased mitochondrial mass and abnormal membrane polarization by flow cytometric analysis with Mitotracker Green and Mitotracker Deep Red probes, respectively (Fig. 1J, K). Thus, these data indicate that defects in mitochondrial functions were associated with the loss of emKCs in obese livers.

### Labile iron deficiency impairs emKC maintenance

**Fig. 2:**
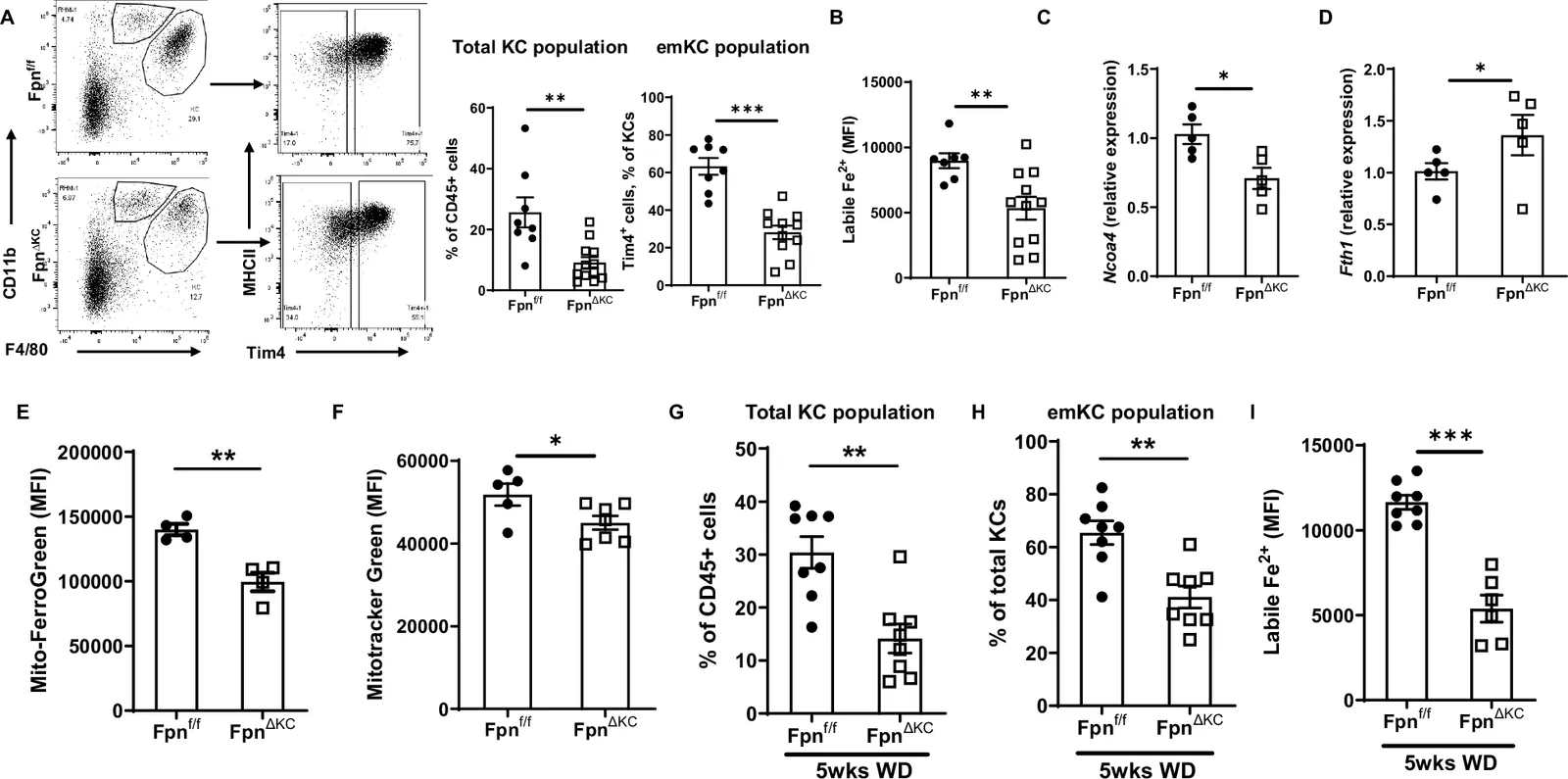
Depletion of ferroportin (Fpn) causes labile iron deficiency in emKCs. **A** The population of total KCs (F4/80^high^CD11b^low^) and emKCs (F4/80^high^CD11b^low^Tim4^+^) of NCD-fed Fpn^f/f^ vs. Clec4fCre^+^Fpn^f/f^ (Fpn^∆KC^) littermates. **B** Effect of *Fpn* depletion on the Fe2+ content of emKCs. The abundance of *Ncoa4* (**C**) and *Fth1* **D** of emKCs after *Fpn* knockout. The levels of mitochondrial Fe2+ (**E**) and mitochondrial mass (**F**) of emKCs after *Fpn* depletion. Total KC population (**G**), emKC proportion (**H**), and emKC Fe2+ levels (**I**) of Fpn^f/f^ vs. Fpn^∆KC^ littermates after 5-weeks WD feeding. Data are presented as mean ± SEM. *P < 0.05, **P < 0.01, ***P < 0.001, Student’s *t* test.

Given that *Fpn* was repressed in emKCs after WD feeding, we generated Clec4fCre^+^Fpn^f/f^ (Fpn^∆KC^) mice, which specifically deplete *Fpn* in KCs (Fig. S2A). As shown in Fig. 2A, lean Fpn^∆KC^ mice had fewer emKC populations than their Fpn^f/f^ littermates. We also observed that *Fpn* depletion resulted in lower levels of labile iron pool in emKCs, compared to lean Fpn^f/f^ littermates (Fig. 2B). Consistently, emKC *Ncoa4* and *Tfrc1* abundance was reduced in Fpn^∆KC^ mice, concomitant with a greater ferritin expression in Fpn^∆KC^ emKCs (Figs. 2C, D and S2B, C). Fpn^∆KC^ emKCs also exhibited a reduction in mitochondrial functions, as evidenced by lower levels of Fe2+ content and mitochondrial mass than Fpn^f/f^ emKCs (Fig. 2E, F). The phenotypes of recruited hepatic macrophages (RHMs; CD11b^high^F4/80^low^) were comparable between lean Fpn^∆KC^ and Fpn^f/f^ littermates (Fig. S2D).

We also assessed the role of *Fpn* in mediating emKC phenotypes in response to WD feeding. After a short period of WD feeding, Fpn^∆KC^ mice had less emKC population than 5-week WD-fed Fpn^f/f^ littermates (Fig. 2G, H). Both Fpn^∆KC^ and WT littermates had comparable RHM population (Fig. S2E). While WD feeding caused a reduction in labile iron content in the emKCs of Fpn^f/f^ littermates, *Fpn* depletion led to a greater level of labile iron deficiency in emKCs in response to WD feeding (Fig. 2I). The expression of ferritin was greater in the emKCs of Fpn^∆KC^ mice than in Fpn^f/f^ littermates after 5-week WD feeding (Fig. S2F, G). In contrast, 5-week WD-fed Fpn^∆KC^ emKCs had a lower abundance of *Tfrc1* than Fpn^f/f^ emKCs (Fig. S2H). *Ncoa4* expression was comparable in emKCs between 5-week WD-fed Fpn^∆KC^ and Fpn^f/f^ mice (Fig. S2I). In addition, WD-fed Fpn^∆KC^ mice exhibited worse mitochondrial defects than Fpn^f/f^ littermates, as shown by lower levels of mitochondrial mass and Fe2+ content in the emKCs of WD-fed Fpn^∆KC^ mice (Fig. S2J, K).

### Labile iron-induced ROS is critical for Trem2 expression in hepatic macrophages

**Fig. 3:**
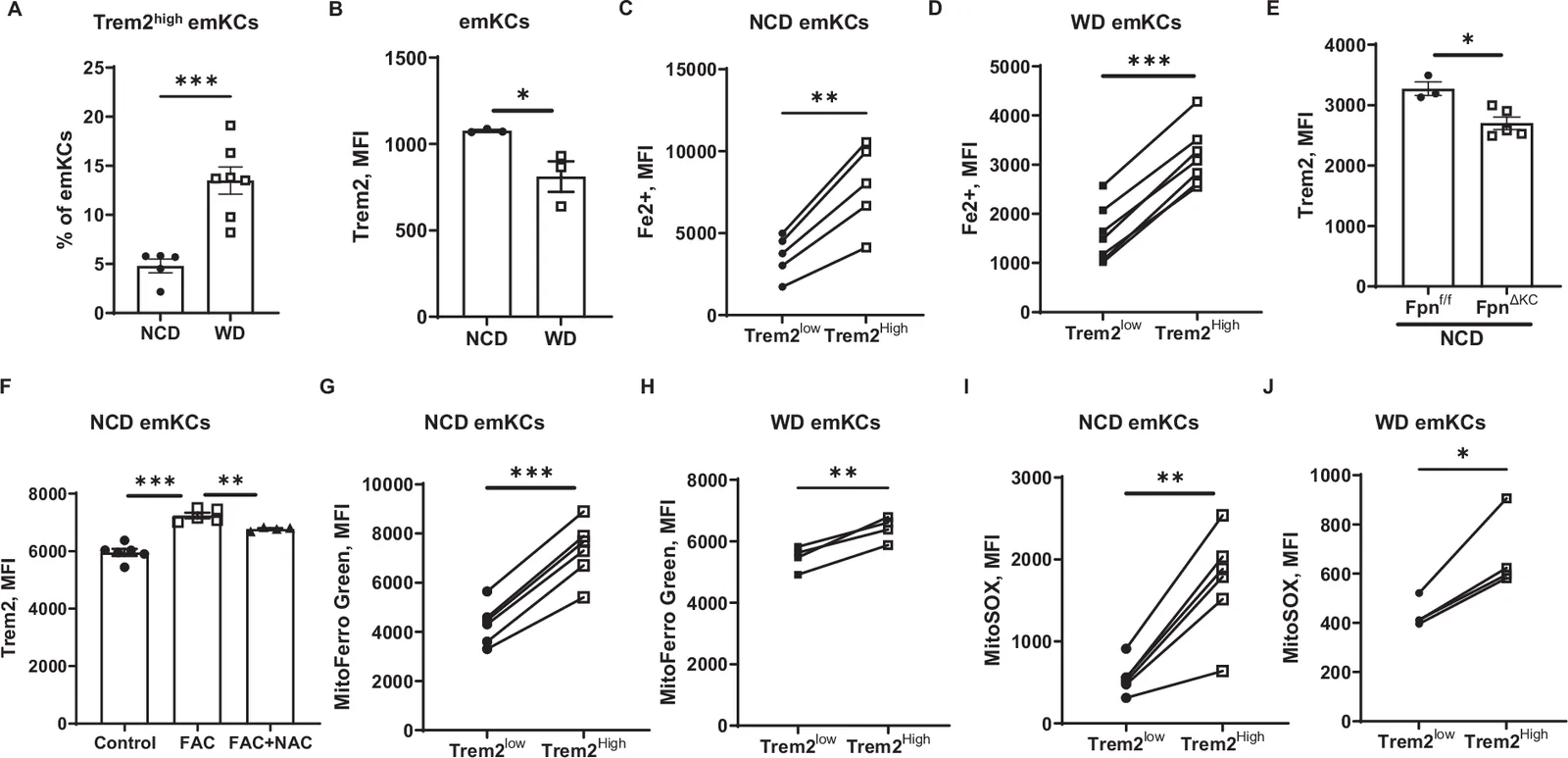
The role of labile iron in mediating Trem2 expression of Kupffer cells. **A** The proportion of Trem2^high^ cells among emKCs after 6wks WD feeding. **B** The abundance of Trem2 in emKCs after 6wks WD feeding. Fe2+ content of Trem2^high^ emKCs vs. Trem2^low^ emKCs of NCD (**C**) or 6wks WD-fed (**D**) WT mice. **E** emKC Trem2 abundance in response to FpnKO. **F** emKC Trem2 abundance after 24 h of FAC and NAC treatment. Comparison of mitochondrial Fe2+ levels between Trem2^high^ emKCs vs. Trem2^low^ emKCs of each NCD (**G**) or 6-weeks WD-fed (**H**) mouse. The levels of ROS production of Trem2^high^ emKCs vs. Trem2^low^ emKCs of each NCD (**I**) or 6-weeks WD-fed (**J**) mouse. Data are presented as mean ± SEM. *P < 0.05, **P < 0.01, ***P < 0.001, Student’s *t* test. **C, D, G–J**, paired t test.

We next assessed the impact of labile iron on liver macrophage functions. Emerging evidence suggests that hepatic macrophage-derived Trem2 is critical to attenuate the development of MASH-related liver abnormalities [23–27]. We found an increase in the proportion of KCs expressing Trem2 in fatty liver, compared to age-matched NCD WT mice (Fig. 3A). However, the abundance of Trem2 of emKCs was lower in fatty liver (Fig. 3B). Interestingly, compared to Trem2^low^ emKCs, Trem2^high^ emKCs contained about 2-3 fold greater levels of Fe2+ in either lean or obese mice (Fig. 3C, D). By comparing KC phenotypes of lean Fpn^∆KC^ and Fpn^f/f^ littermates, we observed that FpnKO-induced Fe2+ reduction was accompanied by lower Trem2 abundance than WT KCs (Fig. 3E). In contrast, in vitro treatment of ferric ammonium citrate (FAC, 100 µM) elevated Trem2 expression of lean KCs (Fig. 3F), supporting the importance of iron supply on upregulating Trem2 expression.

Concomitant with high levels of labile iron pool, Trem2^high^ emKCs were characterized by robust abundance of mitochondrial Fe2 + , compared to Trem2^low^ emKCs (Fig. 3G, H). In response to a high abundance of mitochondrial Fe2 + , ROS production was maintained at greater levels in Trem2^high^ emKCs than Trem2^low^ cells (Fig. 3I, J). To evaluate the importance of ROS on Trem2 expression, lean KCs were treated with FAC and antioxidant NAC in vitro. After 24 h, NAC supplement blunted the effects of FAC on Trem2 expression, suggesting a ROS-dependent mechanism underlying iron-induced Trem2 expression (Fig. 3F).

While previous studies reported that Trem2 is mainly expressed on RHMs [26]. We observed that emKCs expressed greater levels of Trem2 than RHMs in response to WD feeding (Fig. S3A). However, RHMs elevated Trem2 abundance in the obese liver (Fig. S3B). In addition, in WD-fed liver, RHMs were characterized by increased levels of mitochondrial Fe2+ (Fig. S3C). In contrast, RHMs exhibited reduced mitochondrial mass upon WD feeding (Fig. S3D). We also observed that RHMs elevated the expression of ferritin and *Ncoa4* in WD-fed mice (Fig. S3E–I).

### KC-specific *Fpn* depletion exacerbates the development of MASLD/MASH liver abnormalities

**Fig. 4:**
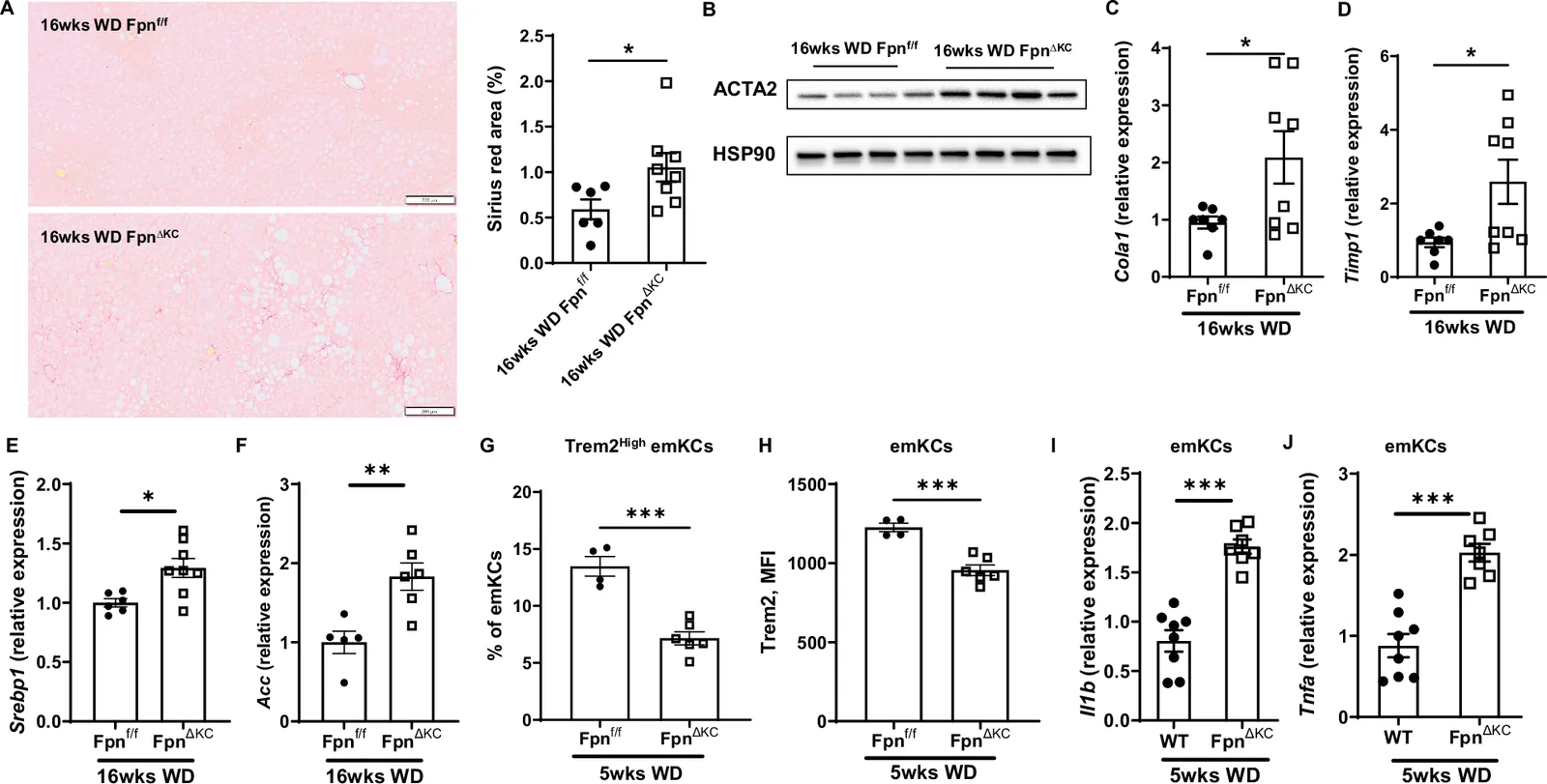
KC-specific *Fpn* depletion promotes the development of MASLD/MASH. **A** Representative images and quantification of Sirius red area in the liver of 16-weeks WD Fpn^f/f^ vs. Fpn^∆KC^ littermates after Sirius Red staining analysis. Scale bar indicates 200 μm. The levels of ACTA2 protein (**B**), *Cola1* (**C**) and *Timp1* (**D**) mRNA in the liver of 16 weeks WD Fpn^f/f^ vs. Fpn^∆KC^ littermates. The expression of *Srebp1* (**E**) and *Acc* (**F**) in the liver of 16-week-old WD Fpn^f/f^ vs. Fpn^∆KC^ littermates. The population of Trem2^high^ emKCs (**G**) and Trem2 abundance (**H**) of 5-week WD-fed Fpn^f/f^ vs. Fpn^∆KC^ littermates. The mRNA levels of *Il1b* (**I**) and *Tnfa* (**J**) of emKCs isolated from 5-week WD-fed Fpn^f/f^ vs. Fpn^∆KC^ littermates. Data are presented as mean ± SEM. *P < 0.05, **P < 0.01, Student’s *t* test.

emKCs exert profound regulation on the development of liver steatosis and fibrosis. Given that *Fpn* depletion promotes WD feeding-induced emKC loss, we also evaluate the impact of emKC loss on the development of liver steatosis and fibrosis by comparing WD-fed Fpn^∆KC^ vs. Fpn^f/f^ littermates. After 16 weeks WD feeding, Fpn^f/f^ mice developed mild liver fibrosis, whereas Fpn^∆KC^ littermates showed more robust liver fibrosis phenotypes (Fig. 4A). In addition, the abundance of ACTA2, which is a fibrosis marker, was greater in the livers of 16 weeks of WD-fed Fpn^∆KC^ mice than that in Fpn^f/f^ littermates (Fig. 4B). The expression of other key genes associated with liver fibrosis development was greater in 16 weeks WD-fed Fpn^∆KC^ livers, compared to Fpn^f/f^ mice (Figs. 4C, D and S4A, B). 16 weeks WD-fed Fpn^∆KC^ mice also developed greater levels of liver steatosis than Fpn^f/f^ littermates, as evidenced by larger size of lipid droplets and higher levels of lipogenic gene abundance in Fpn^∆KC^ liver (Figs. 4A, E, F and S4C, D).

We also found that WD-fed Fpn^∆KC^ mice contained a lower population of Trem2^hi^ emKCs than Fpn^f/f^ littermates (Fig. 4G, H). Consistent with the role of Trem2 in suppressing the expression of proinflammatory cytokines [51, 52]. FpnKO emKCs expressed greater abundance of proinflammatory cytokines *Il1b* and *Tnfa* than Fpn^f/f^ littermates (Fig. 4I, J). In addition, RHM numbers were comparable between 6wks WD-fed Fpn^∆KC^ and Fpn^f/f^ littermates (Fig. S4E). Consistently, 16 weeks WD-fed Fpn^∆KC^ mice had greater levels of proinflammatory cytokines in the liver than Fpn^f/f^ littermates (Fig. S4F, G).

### KC-specific ferritin mutation attenuates MASLD/MASH-related emKC loss and liver abnormalities

Ferritin plays an essential role in storing iron and mediating the levels of the labile iron pool [53, 54]. As obese emKCs showed a reduction in *Fth1* abundance, we established KC-specific *Fth1* knockout (Clec4fCre^+^Fth1^f/f^; Fth1^∆KC^) mice to evaluate the contribution of *Fth1* repression on MASLD/MASH-associated emKC abnormalities (Fig. S5A). Interestingly, *Fth1* mutation had minimal effects on iron phenotypes in emKCs of lean mice, concomitant similar hepatic macrophage phenotypes between lean Fth1^∆KC^ vs. Fth1^f/f^ littermates (Fig. S5B–D). In addition, mitochondrial mass was comparable in the emKCs isolated from lean Fth1^∆KC^ vs. Fth1^f/f^ littermates (Fig. S5E). The expression of key genes associated with iron metabolism in emKCs was not significantly affected by *Fth1* knockout (Fig. S5F). These data suggest a dispensable role of *Fth1* in mediating emKC phenotypes in lean mice.

**Fig. 5:**
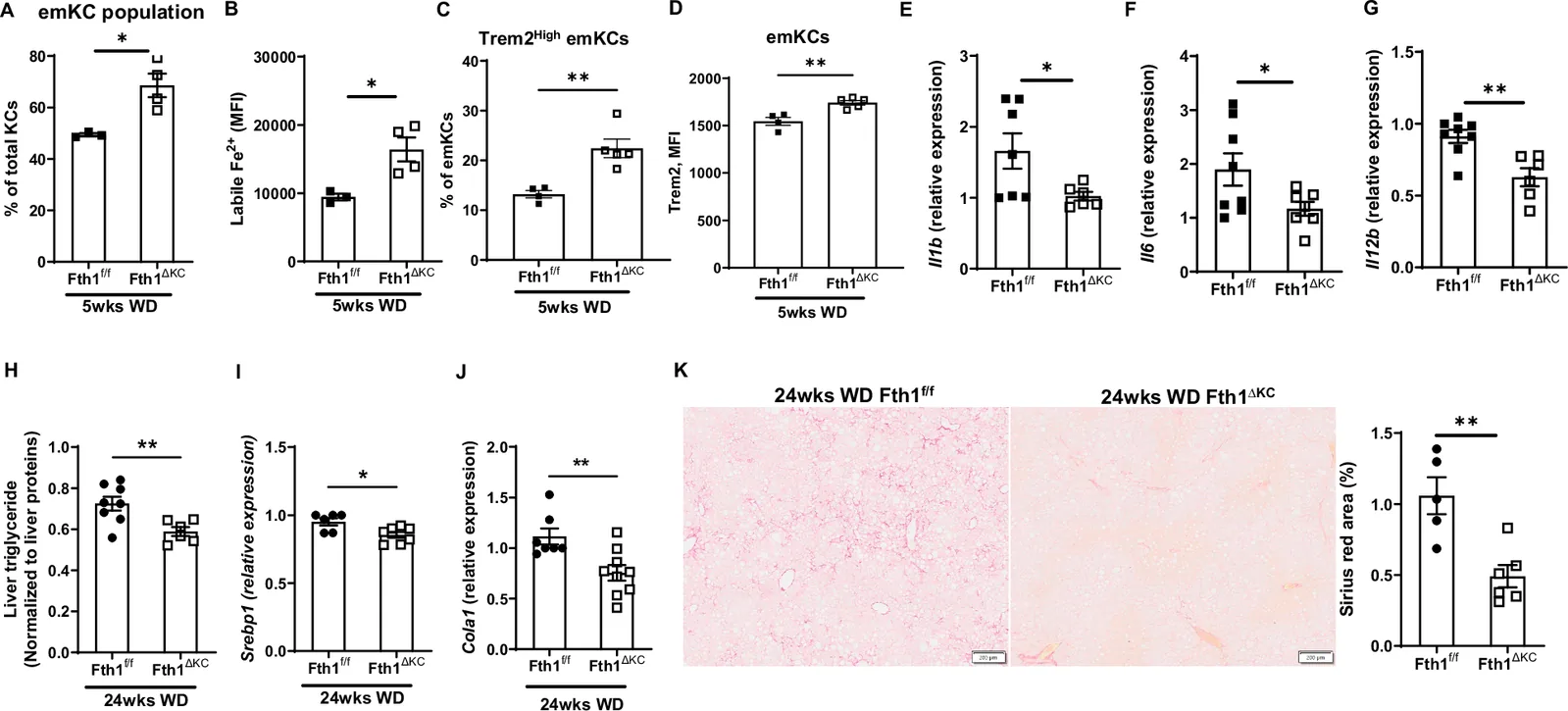
KC-specific ferritin knockout attenuates emKC abnormalities and MASLD/MASH development. emKC population (**A**) and labile iron content (**B**) of Fth1^f/f^ vs. Clec4fCre^+^Fth1^f/f^ (Fth1^∆KC^) littermates after 5-week WD feeding. The population of Trem2^high^ emKCs (**C**) and Trem2 abundance (**D**) of 5-week WD-fed Fth1^f/f^ vs. Fth1^∆KC^ littermates. The mRNA levels of *Il1b* (**E**), *Il6* (**F**), and *Il12b* (**G**) of emKCs isolated from 5-week WD-fed Fth1^f/f^ vs. Fth1^∆KC^ littermates. **H** Liver triglyceride levels of 24wks WD-fed Fth1^f/f^ vs. Fth1^∆KC^ littermates. The expression of *Srebp1* (**I**) and *Cola1* (**J**) in the liver of 24-weeks WD-fed Fth1^f/f^ vs. Fth1^∆KC^ littermates. **K** Representative images and quantification of Sirius red area in the liver of 24-week WD Fth1^f/f^ vs. Fth1^∆KC^ littermates after Sirius Red staining analysis. Scale bar indicates 200 μm. Data are presented as mean ± SEM. *P < 0.05, **P < 0.01, ***P < 0.001, Student’s *t* test.

While *Fth1* depletion did not significantly affect the iron metabolism of the emKCs of lean mice, WD-fed Fth1^∆KC^ mice preserved more emKCs than Fth1^f/f^ littermates (Figs. 5A and S5G). RHM populations were comparable between WD-fed Fth1^∆KC^ and Fth1^f/f^ mice (Fig. S5H). emKCs isolated from WD-fed Fth^∆KC^ mice contained more labile iron content than Fth1^f/f^ littermates (Fig. 5B). Concomitantly, WD-fed Fth^∆KC^ mice had a greater amount of Trem2^hi^ emKCs than Fth1^f/f^ littermates (Fig. 5C, D). In addition, Fth1KO emKCs had a lower abundance of proinflammatory cytokines *Il1b*, *Il6, and Il12b* than that of the emKCs isolated from WD-fed Fth1^f/f^ littermates (Fig. 5E–G).

Concomitant with lower levels of liver inflammation, Fth1^∆KC^ mice showed less liver steatosis than Fth1^f/f^ littermates after 24-weeks WD feeding, as demonstrated by the smaller size of lipid droplets, lower abundance of lipogenic genes and less triglyceride content in Fth1^∆KC^ mice (Figs. 5H–J, K and S5I, J). We also observed that KC-specific *Fth1* knockout led to lower levels of liver fibrosis after 24wks WD feeding (Figs. 5J, K and S5K, L).

### Weight loss normalizes labile iron homeostasis in liver macrophages

**Fig. 6:**
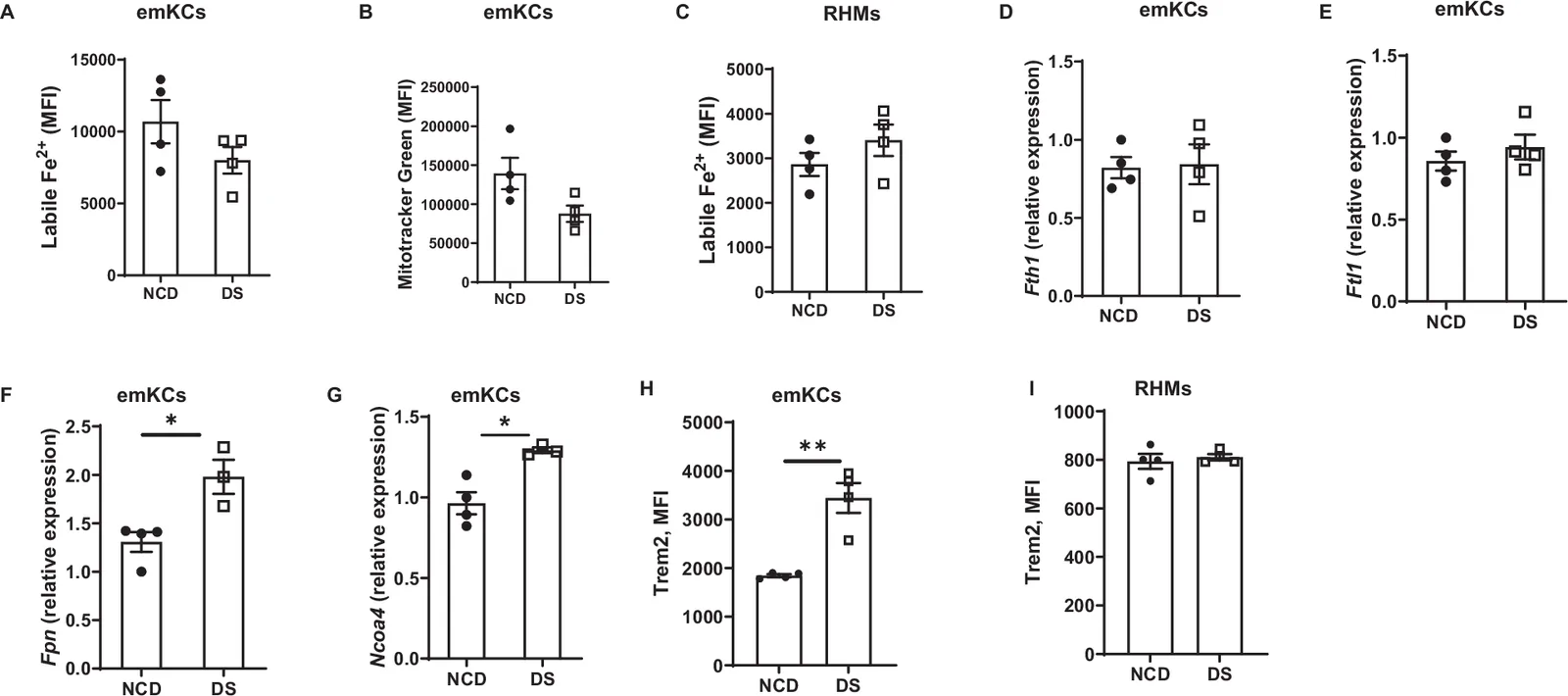
The iron metabolism phenotypes of liver macrophages after weight loss. The levels of labile iron (**A**) and mitochondrial mass (**B**) of emKCs after weight loss by diet switch (DS) to NCD for 6 weeks after 18 weeks of WD feeding. Age-matched NCD WT mice were used as controls. **C** Effect of weight loss on RHM labile iron content. The abundance of key genes associated with emKC iron metabolism. **D–G** after weight loss. Trem2 abundance of emKCs (**H**) and RHMs (**I**) after weight loss. Data are presented as mean ± SEM. *P < 0.05, Student’s *t* test.

Weight loss has been demonstrated as an effective way of resolving liver steatosis and fibrosis. Previous studies, including ours, have shown that hepatic macrophage proportion and functions are also normalized after MASH regression [25, 40]. To induce weight loss, a group of WT mice was fed a WD diet for 18 weeks and then switched to a normal chow diet for another 6 weeks (diet-switch). As expected, the diet switch led to a great reduction in body weight (Fig. S6A). More importantly, weight loss normalized liver macrophage profiles to the levels observed in age-matched NCD WT mice (Fig. S6B, C). emKCs also restored the levels of labile iron content after weight loss, concomitant with similar levels of mitochondrial mass and ROS content to age-matched NCD WT mice (Fig. 6A, B, S6D). The levels of labile iron and mitochondrial mass were comparable in the RHMs isolated from weight loss and age-matched lean mice (Figs. 6C and S6E). In contrast to the repression of iron metabolism activities in the obese liver, emKCs of weight loss mice had comparable ferritin levels to NCD WT mice (Fig. 6D, E). In addition, the expression of *Fpn* and *Ncoa4* was greater in the emKCs of weight loss mice, compared to NCD WT mice (Fig. 6F, G). Concomitant with weight loss-mediated iron homeostasis, emKCs tuned down the levels of proinflammatory activation, as evidenced by comparable abundance of *Il1b* and *Tnfa* in emKCs between weight loss and NCD WT mice (Fig. S6F, G). Similarly, the proinflammatory phenotypes of RHMs were comparable between weight loss mice and age-matched NCD WT mice (Fig. S6F, G). Compared to age-matched lean mice, emKCs elevated Trem2 expression after weight loss, and RHMs had comparable Trem2 abundance (Fig. 6H, I).

## Discussion

Reshaping hepatic macrophage profiles and functions towards proinflammatory phenotypes is one of the drivers for the development of MASLD/MASH. While the liver is the key iron reservoir, how labile iron levels affect hepatic macrophage phenotypes during the process of MASLD/MASH is largely unknown. This study demonstrated that labile iron deficiency caused the loss and functional changes of emKCs in MASLD/MASH liver. MASH emKCs were characterized by a great repression of iron metabolism and cycling. Mutation of iron exporter *Fpn* recaptured the MASLD/MASH-associated emKC labile iron deficiency and mitochondrial defects, accompanied by cell death and dysfunction. These FpnKO-induced emKC abnormalities promoted the development of MASLD/MASH-related liver steatosis and fibrosis. While *Fth1* depletion had minimal effects on emKC phenotypes in lean mice, Fth1^∆KC^ mice attenuated the labile iron deficiency-induced emKC disorders and the development of liver steatosis/fibrosis after WD feeding. Weight loss normalized iron metabolism of hepatic macrophages, concomitant with recovery of hepatic macrophage profile and functions.

It is striking that iron metabolism and the handling capacity of emKCs were markedly repressed in MASLD/MASH liver, resulting in iron deficiency. It has been well-established that emKCs play a crucial role in recycling iron through phagocytosis of dying red blood cells. Previous studies, including ours, have observed a significant decline in the phagocytosis of emKCs in MASLD/MASH liver, which may contribute to the downregulation of iron metabolism and the lack of iron in MASLD/MASH emKCs [12, 21, 37]. In contrast to our observation of emKC iron deficiency in MASLD/MASH liver, Zhang et al. reported that emKCs undergo iron overload and ferroptosis in fatty liver [49]. However, in Zhang et al’s studies, the measurement of labile iron levels by the Phen Green SK probe, which not only detects Fe2+ but also other metal ions, is likely not precise for Fe2+ content. In our studies, we used the BioTracker Far-red probe that specifically interacts with Fe2+ [48]. Consistent with labile iron deficiency, we found that mitochondrial Fe2+ content was greatly reduced in MASLD/MASH emKCs. This reduction was accompanied by profound mitochondrial dysfunction, finally leading to emKC loss.

In lean Fpn^∆KC^ mice, emKCs showed labile iron deficiency, which is likely due to the upregulation of ferritin to seize iron. Consistently, previous studies observed an increase in ferritin in other cell types after mutation of *Fpn* [55]. More importantly, FpnKO-induced labile iron reduction mimicked MASH-associated emKC abnormalities. The loss of emKCs observed in lean Fpn^∆KC^ mice is likely due to the insufficient mitochondrial Fe2+ content-induced mitochondrial mass reduction.

emKCs are one of the iron-enriched cells in the body. However, mutation of *Fth1* had minimal impacts on emKC phenotypes, and the expression of *Ftl1*, *Ncoa4*, *Fpn*, and *Tfrc* was not significantly changed in Fth1KO emKCs. Similarly, early studies have shown that either whole body or cell-specific *Fth1* mutation does not significantly affect cellular iron content [56, 57]. In contrast, in MASLD/MASH liver, *Fth1* knockout attenuated the reduction of labile iron pool and emKC abnormalities, lowering the levels of liver steatosis and fibrosis. Previous studies by Lu et al. have demonstrated that *Fth1* mutation in adipocytes leads to an increase in labile iron content, which promotes energy metabolism [58]. Another study has reported that macrophage-specific *Fth1* knockout attenuates the development of obesity and metabolic disorders [59]. Thus, *Fth1* exerts profound regulation on labile iron supply for emKC maintenance in obesity.

Emerging evidence suggests that Trem2 plays a crucial role in the ability of liver macrophages to regulate liver steatosis and fibrosis [25, 26]. It is interesting that Trem2+ macrophages contained greater levels of labile iron than those cells barely expressing Trem2. The regulation of Fe2+ levels on Trem2 expression is further supported by the observation from KC-specific FpnKO or Fth1KO mice. Concomitant with high Fe2+ content, Trem2+ cells produced high levels of ROS. In contrast, ROS chelation blunted the ability of iron enrichment to induce Trem2 expression, suggesting a ROS-dependent mechanism underlying Trem2 expression. Consistent with the crucial role of Trem2 in MASLD/MASH development, FpnKO-induced Trem2 downregulation was accompanied by a quicker development of liver steatosis and fibrosis. In contrast, elevation of Trem2 may contribute to the attenuation of MASLD/MASH liver phenotypes of WD-fed Fth1KO mice.

Several studies have reported the impacts of weight loss on immune cell phenotypes [25, 40, 60]. Our previous studies have shown the critical role of normalization of emKC complement immune functions in mediating weight loss-associated metabolic recovery [40]. Consistently, we observed that weight loss restored hepatic macrophage profiles to the levels of age-matched lean mice. More importantly, iron metabolism was also normalized in emKCs after weight loss. Previous studies have highlighted the role of hyperlipidemia in inducing macrophage abnormalities [14, 26]. Thus, switching to NCD feeding lowers circulating lipid levels, which likely contributes to restoring hepatic macrophage phenotypes. In addition, Ganguly et al. showed that liver macrophage Trem2 is required for the weight loss-mediated MASH regression [25].

In summary, our findings highlight the crucial role of labile iron homeostasis in emKC maintenance in MASLD/MASH liver. Thus, interventions focusing on normalizing emKC labile iron homeostasis could lead to a promising therapeutic approach for MASLD/MASH.

## Materials and methods

### Mice

All animal studies complied with approved protocols by the University of California, San Diego Research Guidelines for the Care and Use of Laboratory Animals. The mice were housed at 22 °C under a 12-h light/dark cycle and *ad libitum* access to a normal chow diet (TestDiet 5TJS; NCD) and water. To induce MASLD/MASH phenotypes, mice were fed an AIN-76A Western Diet (TestDiet 5342; WD) containing 40% fat calories and 0.2% cholesterol up to 24 weeks. Clec4fCre mice were transferred from Dr. Christopher Glass lab (University of California, San Diego). Fth1 flox mice were provided by Dr. Abolfazl Zarjou (University of Alabama at Birmingham). Fpn flox mice were provided by Dr. Yatrik Shah (University of Michigan). All the mice were housed in the University of California, San Diego animal facility. Clec4fCre mice were crossed with either Fpn^f/f^ or Fth1^f/f^ mice to induce Kupffer cell-specific *Fpn* (Fpn^ΔKC^) or *Fth1* (Fth1^ΔKC^) knockout, respectively.

### Isolation of non-parenchymal cells from the liver

The mice were under anesthetized, and the liver was perfused via the vena cava with 50 mL of pre-warmed (37 °C) calcium- and magnesium-free HBSS supplemented with 0.25 mM EGTA and 10 mM HEPES, followed by 30 mL of warm (37 °C) HBSS containing calcium and magnesium, 10 mM HEPES, and 1 mg/mL collagenase D. The liver tissue was then dissociated by stirring with a scraper in PBS with 2% FBS, and filtered through a 100 μm cell strainer. Parenchymal cells were removed through a series of low-speed centrifugations (50 × *g*, 80 × *g*, and 100 × *g*, each for 5 min at room temperature; an additional 100 × g step was included for obese livers). The supernatant was collected and centrifuged (1000 × *g*, 10 min at room temperature) to pellet the cells. Non-parenchymal cells were further purified using a 28% OptiPrep density gradient centrifugation (1400 × *g*, 25 min, low acceleration, no brake, 4 °C), with the interphase layer harvested afterward. Red blood cells were lysed using 1X RBC Lysis Buffer before staining for flow cytometry.

### Flow cytometry analysis and cell sorting

Before staining, cells were incubated with CD16/32 antibody for 5 min at room temperature to block non-specific binding of antibodies. To analyze and sort out hepatic macrophages, cells were stained with LIVE/DEAD Fixable Aqua Dead Cell dye (Cat # L34966, Thermofisher) and cell surface antibodies for 30 min at 4 °C, and then analyzed with SONY MA900 Cell Sorter. Hepatic macrophages were classified and sorted into three types: emKCs (Live^+^CD45^+^F4/80^high^CD11b^low^Tim4^+^; Cat# 123114, 101236, 130020, Biolegend), KC-like macrophages (Live^+^CD45^+^F4/80^high^CD11b^low^Tim4^-^), and RHMs (Live^+^CD45^+^F4/80^low^CD11b^high^). The labile iron levels were evaluated with BioTracker Far-red labile iron dye (Cat # SCT037, Sigma). To examine iron or mitochondrial alterations, the cells were stained with the probes and cell surface antibodies for 40 min at room temperature and analyzed by MA900.

### Western blot analysis

The liver was homogenized in 1X RIPA buffer supplemented with Protease and Phosphatase Inhibitor Cocktail. Protein concentrations were quantified using the DC Protein Assay (Bio-Rad). The samples were run on Mini-PROTEAN TGX gels (Bio-Rad) and transferred to PVDF membranes. Membranes were blocked with 5% nonfat milk in TBST for 1 h at room temperature, followed by overnight incubation with primary antibodies at 4 °C. After three 10-min TBST washes, membranes were incubated with secondary antibodies for 1 h at room temperature and washed again with TBST three times. Protein bands were imaged using SuperSignal™ West Pico PLUS Chemiluminescent Substrate (Thermo Fisher Scientific) with Image Lab software (Bio-Rad).

### Gene expression analysis

Total RNAs were extracted from sorted cells or tissue using TRIzol reagent. High-Capacity cDNA Reverse Transcription Kit was used to synthesize cDNA according to the manufacturer’s instructions. qPCR was performed with 10 μL of reaction using PerfeCTa SYBR Green FastMix ROX on a QuantStudio 3 Real-Time PCR System. The relative expression levels of genes were normalized to β-actin or Rpl13a.

### Measurements of triglycerides

Hepatic triglyceride levels were quantified using the Triglyceride Colorimetric Assay kit (Cayman), following the manufacturer’s instructions, with normalization to the total protein content.

### Quantification and statistical analysis

Samples were blinded whenever deemed appropriate and applicable. Sample description and identification were not available to the core lab personnel during data plotting and analyses. No samples or data were excluded from the study for statistical purposes. Each in vitro experiment was independently performed in duplicate or triplicate to ensure reproducibility. Group sizes of 5 mice or greater were sufficient to reach a statistical power of at least 80%. Animals were assigned at random to treatment groups for all studies. The tests used for statistical analyses are described in the figure legends. All sample numbers are given in the figure legends. To determine whether the means of two groups are statistically different from each other, unpaired two-tailed Student’s *t*-tests were used for statistical analyses. All data passed the normality test using Prism 10 software (GraphPad software v10.0; Prism, La Jolla, CA). *P* values of 0.05 or less were considered statistically significant.

## Supplementary information

Supplementary figures

uncropped Western blot images

## Data Availability

The dataset generated during and/or analysed during the current study is available from the corresponding author on reasonable request.
