# Supplementary material for: Labile iron starvation in embryonic Kupffer cells aggravates MASH via mitochondrial failure and macrophage dysfunction
Source: Cell Death Dis. 2026 Jun 18;17(1):879. doi: 10.1038/s41419-026-08985-7 (PMC13654985; doi:10.1038/s41419-026-08985-7)
Supplement: Supplementary file 1 [file 41419_2026_8985_MOESM1_ESM.pdf]

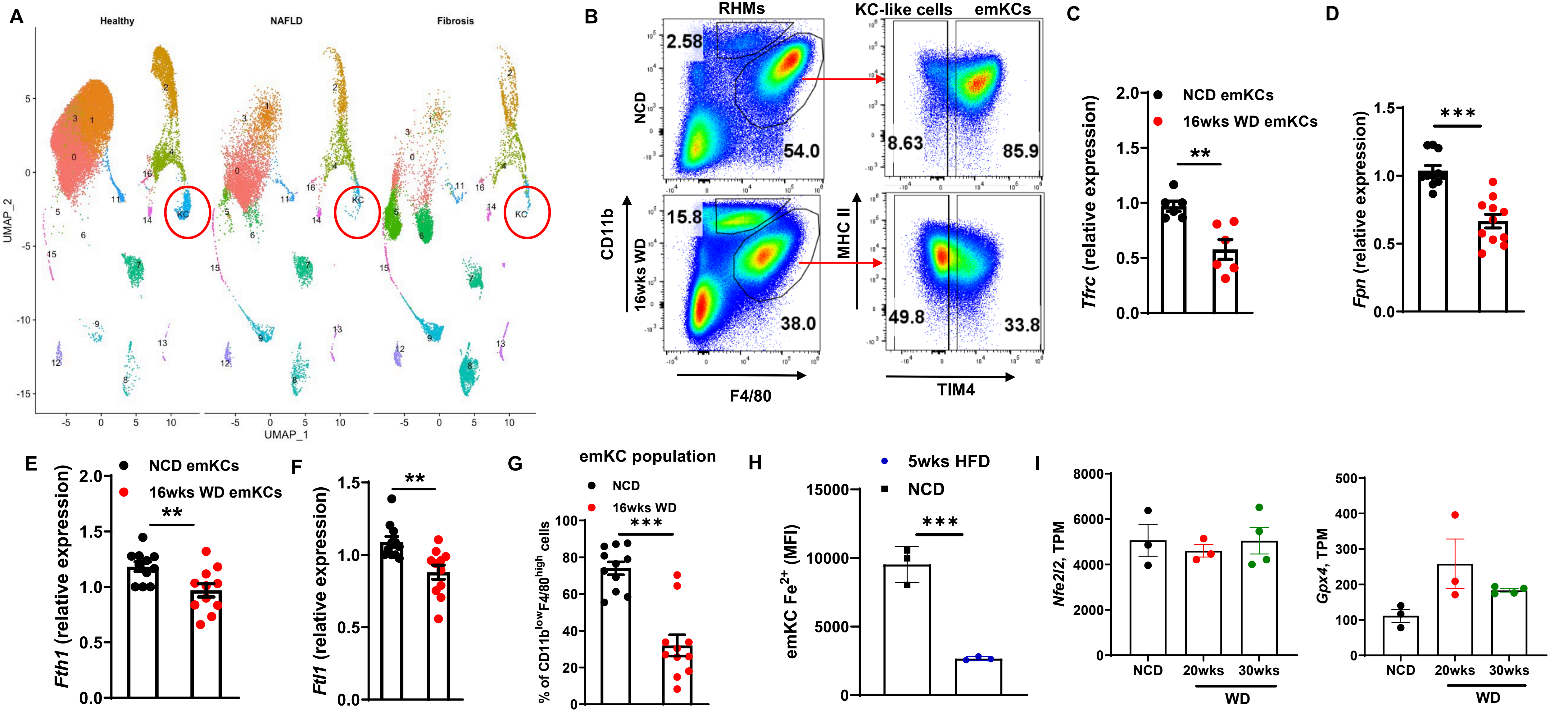

Figure S1. The iron metabolism phenotypes in embryonic Kupffer cells (emKCs) in MASLD/MASH liver. (A) UMAP showing different cell clusters including Timd4+Marco+CD68+ emKCs (highlighted in red circle) in human livers. (B) Flow cytometric analysis (FACS) of RHMs (CD11b^{high}F4/80^{low}), emKCs (TIM4^{+}CD11b^{low}F4/80^{high}), and KC-like cells (TIM4^{-}CD11b^{low}F4/80^{high}) of mouse livers. (C-F) The expression of iron metabolism genes in the emKCs of 16wks Western diet (WD)-fed WT vs. age-matched normal chow diet (NCD)-fed WT mice. (G) emKC population after 16wks WD feeding. (H) emKC labile iron levels after 5wks high-fat diet (HFD) feeding. (I) The expression of *Nfe2l2* and *Gpx4* in KCs after WD feeding. TPM, transcript per kilobase million. Data are presented as mean ± SEM. ***P*<0.01, ****P*<0.001, Student's t-test.

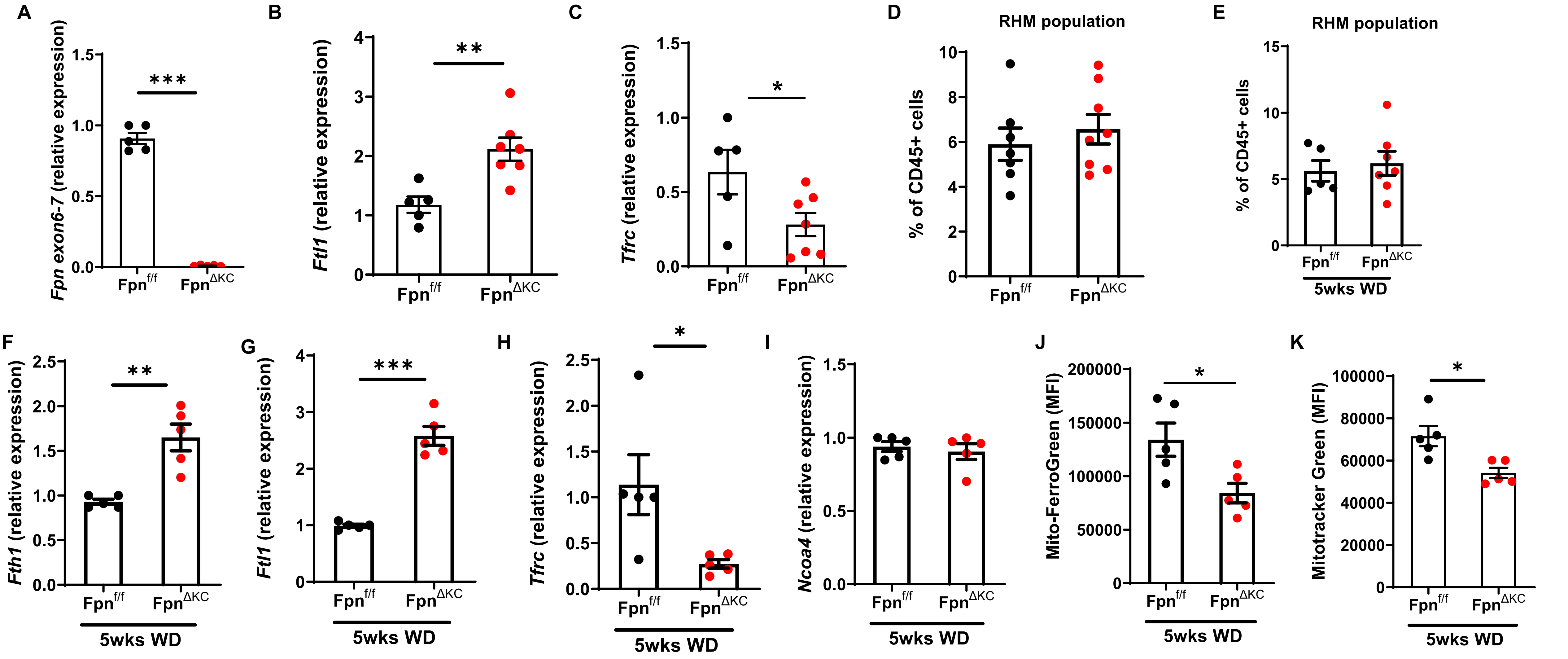

Figure S2. The importance of labile iron homeostasis in emKC maintenance. The mRNA levels of *Fpn* (A), *Ftl1* (B), *Tfrc1* (C) in emKCs isolated from NCD-fed $Fpn^{f/f}$ vs. $Clec4fCre^{+}Fpn^{f/f}$ ($Fpn^{\Delta KC}$) littermates. RHM population of lean (D) or 5wks WD (E)-fed $Fpn^{f/f}$ vs. $Fpn^{\Delta KC}$ littermates. (F-I) The mRNA levels of key genes associated with iron metabolism in the emKCs isolated from 5wks WD-fed $Fpn^{f/f}$ vs. $Fpn^{\Delta KC}$ littermates. The levels of mitochondrial Fe2+ (J) and mitochondrial mass (K) of the emKCs isolated from 5wks WD-fed $Fpn^{f/f}$ vs. $Fpn^{\Delta KC}$ littermates. Data are presented as mean ± SEM. *P<0.05, **P<0.01, ***P<0.001, Student's t-test.

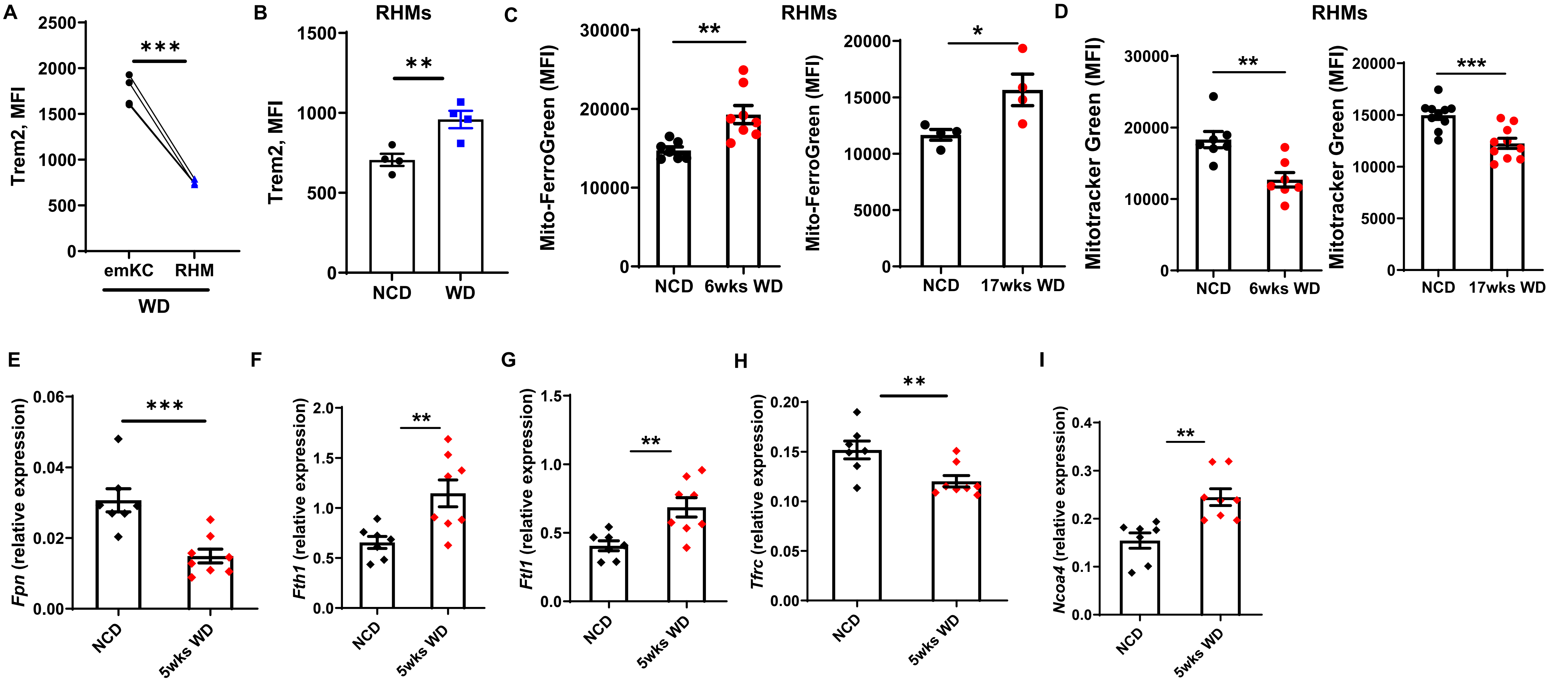

Figure S3. Effect of labile iron homeostasis on Trem2 expression. (A) Comparison of Trem2 abundance between emKCs and RHMs after 6wks WD feeding. (B) RHM Trem2 abundance after 16wks WD feeding. The levels of mitochondrial Fe^{2+} (C) and mitochondrial mass (D) in RHMs after WD feeding. NCD mice were age-matched WT mice. (E-I) The expression of key genes associated with iron metabolism in RHMs after 5wks WD feeding. Data are presented as mean ± SEM. *P<0.05, Student's t-test.

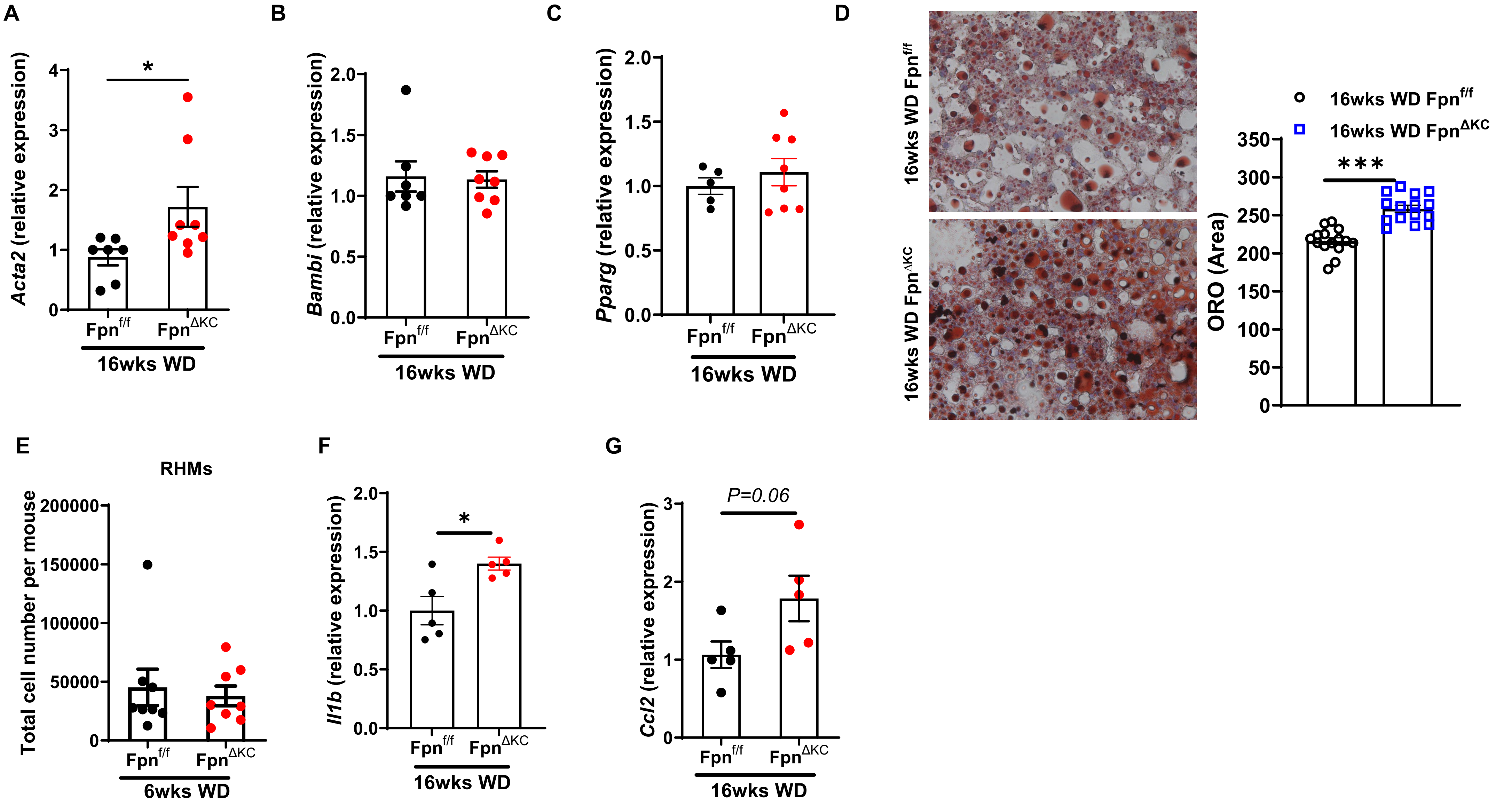

Figure S4. The liver phenotypes of KC-specific *Fpn* knockout mice after WD feeding. The mRNA levels of *Acta2* (A), *Bambi* (B), and *Pparg* (C) in the liver of $Fpn^{f/f}$ vs. $Fpn^{\Delta KC}$ littermates after 16wks WD feeding. D, Oil red O (ORO) staining analysis of liver lipid content of 16wks WD-fed $Fpn^{f/f}$ vs. $Fpn^{\Delta KC}$ littermates. E, Total cell number of RHMs the liver of $Fpn^{f/f}$ vs. $Fpn^{\Delta KC}$ littermates after 6wks WD feeding. The expression of *Il1b* (F) and *Ccl2* (G) in the liver of $Fpn^{f/f}$ vs. $Fpn^{\Delta KC}$ littermates after 16wks WD feeding. Data are presented as mean ± SEM. **P*<0.05, Student's t-test.

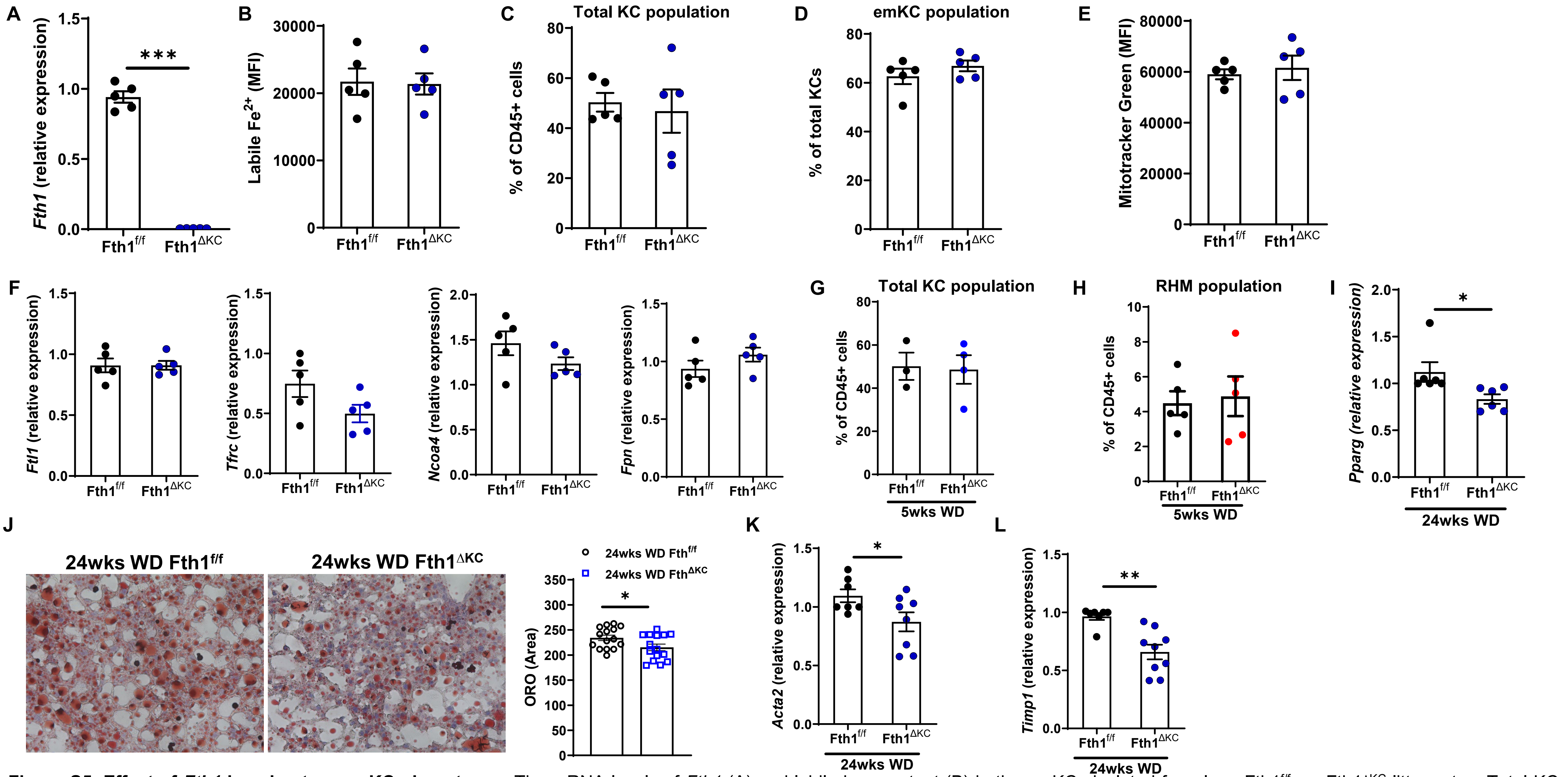

Figure S5. Effect of *Fth1* knockout on emKC phenotypes. The mRNA levels of *Fth1* (A) and labile iron content (B) in the emKCs isolated from lean $Fth1^{f/f}$ vs. $Fth1^{\Delta KC}$ littermates. Total KC population (C) and emKC proportion (D) in the liver of lean $Fth1^{f/f}$ vs. $Fth1^{\Delta KC}$ littermates. (E) The levels of mitochondrial mass in the emKCs of lean $Fth1^{f/f}$ vs. $Fth1^{\Delta KC}$ littermates. (F) Effect of *Fth1* knockout on the expression of iron metabolism gene abundance in emKCs. Total KC (G) and RHM (H) populations in the liver of $Fth1^{f/f}$ vs. $Fth1^{\Delta KC}$ littermates after 5wks WD feeding. The mRNA levels of *Pparg* (I), *Acta2* (K), and *Timp1* (L) in the liver of 24wks WD-fed $Fth1^{f/f}$ vs. $Fth1^{\Delta KC}$ littermates. J, Oil Red O staining analysis of liver lipid content. Data are presented as mean ± SEM. **P*<0.05, ***P*<0.01, ****P*<0.001, Student's t-test.

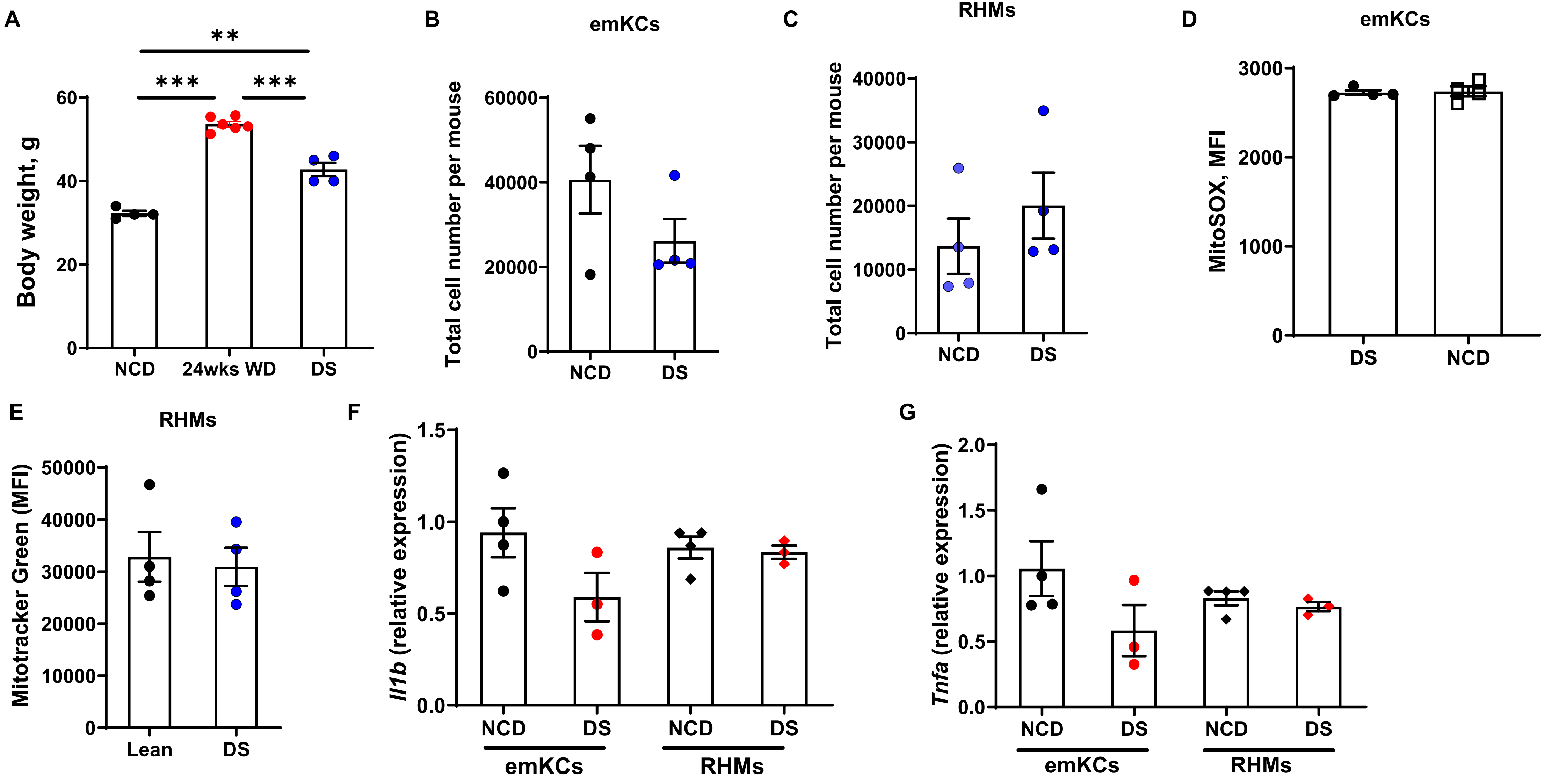

Figure S6. Effect of weight loss on hepatic macrophage iron metabolism phenotypes. (A) Body weight of 18wks WD-fed WT mice after switching to NCD feeding for 6wks. Total cell number of emKCs (B) and RHMs (C) after weight loss. Age-matched NCD-fed WT mice were used as controls. D, emKC mitochondrial ROS content after weight loss. E, The levels of mitochondrial mass of RHMs after weight loss. The mRNA levels of *Il1b* (F) and *Tnfa* (G) of liver macrophages after weight loss. Data are presented as mean ± SEM.
